# A Compositional Calibration Framework for Multi-Channel Functional Electrical Stimulation Enabling Hand Gesture Generation

**DOI:** 10.3390/bioengineering13060701

**Published:** 2026-06-18

**Authors:** Elena Stefanel, Nicolò Landra, Andrea Prestia, Fabio Rossi, Andrea Mongardi, Paolo Motto Ros, Danilo Demarchi

**Affiliations:** Department of Electronics and Telecommunications, Politecnico di Torino, 10129 Turin, Italy; elena.stefanel@studenti.polito.it (E.S.); andrea.prestia@polito.it (A.P.); fabio.rossi@polito.it (F.R.); andrea.mongardi@polito.it (A.M.); paolo.mottoros@polito.it (P.M.R.); danilo.demarchi@polito.it (D.D.)

**Keywords:** functional electrical stimulation, hand rehabilitation, multi-channel stimulation, calibration

## Abstract

The application of functional electrical stimulation (FES) to restore hand motor function remains challenging due to the difficulty of calibrating multi-channel stimulation to produce coordinated finger movements. This study proposes a compositional FES calibration framework to customize the stimulation of isolated finger actions and enable their combination into functional hand gestures. The proposed method was validated through a two-session experimental study involving thirteen participants. In the first session, subject-specific stimulation sites and parameters were identified for eight individual finger movements using a structured spatial grid defined over the forearm. The second session, conducted on a subset of five participants, evaluated the generation of seven hand gestures derived from combinations of the isolated movements. Results showed that ten of the thirteen participants achieved at least six movements, while three participants successfully elicited all targeted motions. Successfully elicited movements were generally well isolated, although thumb and ring/little finger extensions proved more difficult to isolate. The second session demonstrated that individually calibrated finger activations can be combined to produce coordinated multi-finger movement patterns, with average finger excursions matching the expected motions. Overall, these preliminary results support the use of compositional calibration strategies to achieve functional multi-finger control with multi-channel FES.

## 1. Introduction

The human hand is a highly sophisticated anatomical structure that serves as an essential tool for a wide range of daily activities. It exhibits over 20 degrees of freedom (DoF), controlled by more than 30 muscles [1,2,3], enabling a remarkable level of dexterity, precision, and versatility. Motor impairment of hand function has a profound impact on quality of life, limiting an individual’s autonomy and ability to interact with the surrounding environment [4,5]. Neurological conditions such as stroke and spinal cord injury affect millions of people worldwide [6,7] and often result in paralysis or severe motor impairment, particularly in the upper limbs [8,9].

Rehabilitation using Functional Electrical Stimulation (FES) has emerged as an important therapeutic approach for promoting motor recovery [4]. FES is widely used to elicit muscle contractions through low-energy electrical pulses, thereby facilitating the restoration of functional movements [10,11,12].

Electrical stimulation is primarily characterized by three parameters that influence the stimulation outcome: frequency, current intensity, and pulse width. Frequency determines the rate at which stimulation pulses are delivered and plays a key role in shaping the temporal summation of muscle responses. In FES, higher frequencies (typically 20 Hz to 50 Hz) are commonly required to achieve smooth and sustained contractions compared to the lower firing rates observed under physiological conditions (around 6 Hz to 8 Hz), due to the synchronous recruitment of motor units [10].

Current intensity (CI) defines the amplitude of the stimulation and primarily affects the number of recruited motor units. Typical FES pulses do not exceed 100 mA [12]. Lower stimulation intensities have been suggested to preferentially promote central nervous system adaptations [13].

Pulse width (PW) refers to the duration of each stimulation pulse and generally ranges between 200 μs and 500 μs. It contributes to motor unit recruitment alongside current amplitude, with larger pulse widths leading to stronger contractions and enabling activation of deeper tissues [14]. The need to appropriately balance these parameters becomes even more critical in FES systems intended for functional hand rehabilitation, where selectivity and adaptability are essential. Consequently, identifying suitable combinations of stimulation parameters and electrode locations represents a key challenge, motivating the development of effective calibration strategies.

FES calibration becomes even more challenging when targeting fine, dexterous hand movements, due to the complex neuromuscular organization of the forearm and the inherent difficulty of selectively activating individual muscles using surface stimulation [15,16,17]. Early wearable neuroprosthetic systems, such as the Bionic Glove [18] and the NESS Handmaster [19], demonstrated the feasibility of restoring basic hand functions (e.g., grasp and release) using surface electrodes and relatively simple control strategies. However, their control capabilities were largely limited to coarse motor tasks, with a restricted ability to modulate individual finger movements or adapt stimulation to varying task demands.

More recent approaches have sought to enhance functional performance by complementing FES with external assistive devices, including soft exoskeleton gloves, robotic platforms, and hybrid rehabilitation systems [20,21,22]. While these hybrid solutions improve task-level performance and robustness, they often compensate for, rather than address, the fundamental limitations of FES in achieving selective and dexterous finger control.

At the core of these limitations lies the difficulty of selectively activating individual finger movements using surface stimulation. Hand dexterity relies on the coordinated contraction of intrinsic and extrinsic muscles. While stimulation of intrinsic muscles can produce more selective effects, their functional contribution is typically limited to metacarpophalangeal (MCP) flexion combined with interphalangeal (IP) extension [23]. In contrast, extrinsic muscles located in the forearm are highly redundant and anatomically coupled, with multiple tendons spanning several joints and fingers. As a result, electrical stimulation delivered through surface electrodes tends to spread across adjacent motor units, leading to unintended co-activation of muscles and reduced selectivity [24,25,26].

This limitation is further exacerbated by electrode displacement during movement, variations in tissue properties, and substantial inter- and intra-subject variability in neuromuscular response [15,16]. Additionally, stimulation intensity is often constrained by user comfort, limiting the achievable torque and further reducing control precision [22].

To address these challenges, recent research has focused on improving stimulation selectivity and personalization. Approaches include the use of high-density electrode arrays, which increase the spatial resolution of stimulation and enable more flexible activation patterns [27,28]. Systematic investigations of electrode placement and motor point characterization have also been conducted to better understand the relationship between stimulation location and functional output [15,24,25]. In parallel, optimization-based and machine learning-driven calibration methods have been proposed to automatically identify subject-specific stimulation parameters and electrode configurations, reducing reliance on manual tuning [17].

Complementary efforts have explored advanced control strategies, including multipoint stimulation and closed-loop neuromuscular control using kinematic or force feedback [26,29,30,31]. These methods aim to regulate functional outputs such as finger position or grasp force in real time, partially mitigating the variability and nonlinearity of the neuromuscular response. Dedicated devices, such as multi-channel FES gloves and selective stimulation systems, have demonstrated the ability to induce controlled movements of individual digits under constrained experimental conditions [26,32].

Collectively, these studies demonstrate that selective activation of individual fingers is achievable to some extent. However, they also highlight a fundamental trade-off: increasing stimulation selectivity typically requires more complex hardware (e.g., dense electrode arrays, multi-channel stimulators) and more sophisticated calibration procedures. Identifying optimal stimulation sites and parameters across large electrode sets remains computationally demanding and time-consuming, often requiring expert supervision [17]. Consequently, despite advances in automation and control, the combined challenges of selectivity, scalability, and usability continue to limit the practical deployment of FES-based systems for dexterous hand rehabilitation, particularly in unsupervised or home-based settings.

Additionally, while several studies have demonstrated selective activation of individual fingers and limited multi-finger patterns using high-density or multipoint stimulation, these approaches remain constrained to predefined or low-dimensional movements, and do not yet enable general dexterous hand gesture control.

To address these limitations, this work proposes a compositional calibration framework for multi-channel FES tailored to low-cost systems with a limited number of stimulation channels. Instead of relying on high-density electrode arrays, a spatial grid is defined over the forearm to systematically explore and identify effective stimulation sites within a constrained search space. Although this approach does not provide the same spatial resolution as dense arrays, it preserves the possibility of fine manual adjustment of electrode locations and facilitates stimulation-site recovery across sessions while maintaining a simpler hardware architecture.

The proposed method enables the configuration of up to eight stimulation channels to elicit selective finger activations and reproducible multi-finger patterns. Building on this, complex hand gestures are generated by combining individually calibrated finger movements, following a compositional approach that decouples calibration from gesture synthesis. The framework is evaluated in healthy participants as a pilot study to investigate:

- The feasibility of identifying stimulation electrode placements and parameters capable of eliciting isolated finger movements through a grid-based calibration procedure.
- The feasibility of recovering previously identified stimulation sites and parameters after electrode removal and grid reconstruction.
- The feasibility of generating dexterous hand gestures through the composition of individually calibrated finger movement primitives.

By leveraging structured spatial sampling and modular activation strategies, the framework aims to achieve a favorable trade-off between stimulation selectivity, system simplicity, and usability. This makes it a promising candidate for practical deployment in wearable and potentially home-based rehabilitation scenarios, where minimizing hardware complexity and calibration burden is essential.

## 2. Compositional Calibration of FES for Complex Hand Gestures

Complex functional hand movements can be modeled as compositions of fundamental finger motions with precise temporal coordination. For example, finger counting can be represented as the combination of elementary movements such as flexion/extension and thumb opposition. Accordingly, the calibration of FES parameters and stimulation sites to elicit complex gestures can be achieved by decomposing each gesture into its constituent motions and iteratively calibrating the stimulation required for each component.

**Figure 1:**
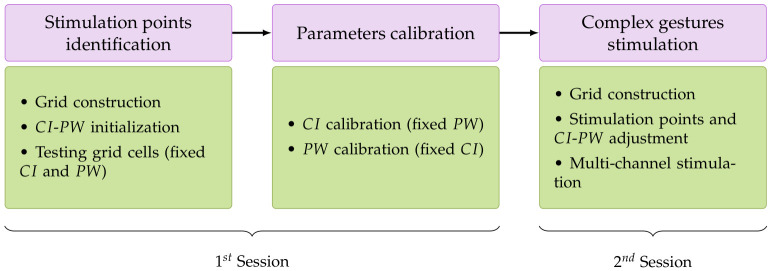
Block diagram of the multi-channel hand FES protocol. An initial calibration session identifies the optimal current intensity (CI), pulse width (PW) and electrode layout. In subsequent sessions, stimulation sites and parameters are refined to elicit complex hand gestures.

Building on this compositional approach, the proposed calibration protocol provides a modular framework for multi-channel FES therapy in complex hand gesture rehabilitation. The protocol consists of two distinct phases performed during the initial calibration session. The resulting electrode layout and optimized stimulation parameters can then be adjusted and reused in subsequent sessions for the elicitation of dexterous hand gestures, as illustrated in Figure 1.

To account for inter-subject variability in anatomical dimensions and stimulation response, and to ensure consistent electrode placement across sessions, stimulation site identification is standardized using a grid-based system. As described in Section 2.1, the grid can be easily traced on the patient’s skin at the beginning of each session, leveraging anatomical landmarks and anthropometric measurements.

Once the optimal electrode placement for each finger movement is established, the pulse width (PW) and current intensity (CI) are calibrated individually, as described in Section 2.3. In this work, the stimulation frequency is kept fixed at 40 Hz, which is commonly used to achieve tetanic contractions [11,31]. Stimulation is delivered using doublet pulses with a 12 ms interpulse interval. This strategy was adopted empirically during preliminary protocol development based on participant comfort and consistent muscle activation.

**Figure 2:**
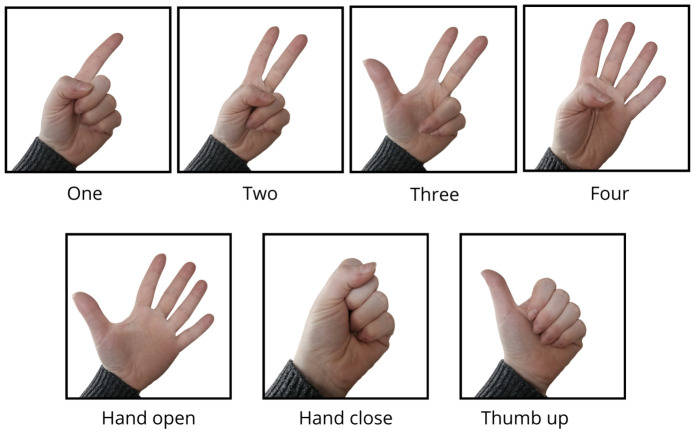
Representative hand gestures elicited in the study.

Figure 2 illustrates seven gestures used for finger counting that were employed to evaluate the proposed calibration framework. These gestures result from the combination of eight distinct finger movements: thumb extension (TE), thumb opposition (TO), index flexion (IF), index extension (IE), middle flexion (MF), middle extension (ME), ring and little finger flexion (RLF), and ring and little finger extension (RLE). These isolated motions were elicited by electrically stimulating the extrinsic muscles flexor digitorum superficialis, extensor digitorum communis, and extensor pollicis longus. The TO movement was elicited by stimulating the intrinsic muscle opponens pollicis.

### 2.1. Grid System Construction

The grid is designed to (i) cover the skin areas over the targeted muscles, (ii) be quick and easy to replicate across sessions, and (iii) adapt to different forearm sizes. Grid scaling is standardized using three anthropometric measurements: wrist circumference ($c_{w}$), elbow circumference ($c_{e}$), and forearm length ($l_{fa}$), defined as the distance from the radial styloid process to the lateral epicondyle.

**Figure 3:**
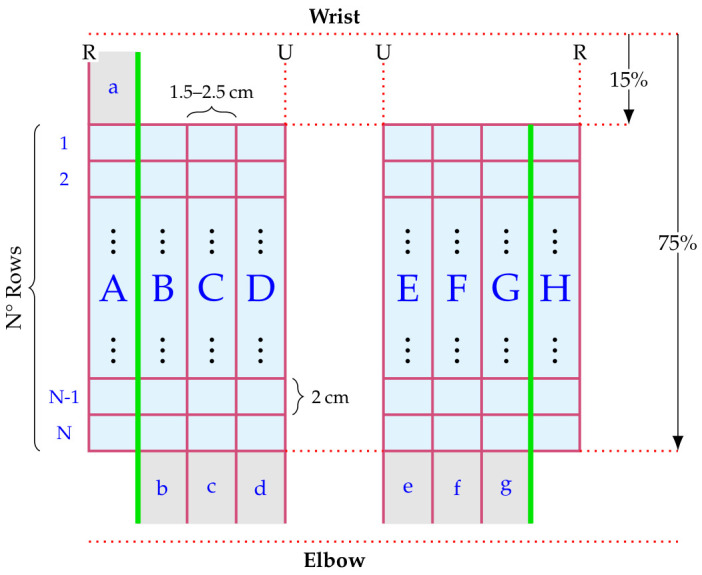
Positioning of stimulation electrodes relative to the grid (light blue) on the posterior (**left**) and anterior (**right**) forearm. The red dashed lines indicate the wrist (**top**) and elbow (**bottom**) levels. U and R denote the ulnar and radial sides, respectively, assuming a right forearm. Grid rows are spaced at 2 cm, with transverse spacing varying along the forearm. Capital letters index the grid columns, while numbers from 1 to *N* index the rows. Lowercase letters indicate return electrode placements (gray cells).

As shown in Figure 3, the grid system consists of two grids drawn with a dermatographic marker on the anterior and posterior forearm, spanning approximately 60% of the $l_{fa}$. Each grid comprises four columns aligned with the forearm’s longitudinal axis, roughly corresponding to the four fingers excluding the thumb. The number of rows (#Rows) in the transverse direction depends on $l_{fa}$ and may therefore vary across subjects.

To facilitate consistent identification of stimulation sites across sessions, each cell is labeled with a capital letter and a number indicating its column and row: A–D for the posterior forearm, encoding columns from the radial to the ulnar side, and E–H for the anterior forearm, following the same ulnar-to-radial orientation. This is combined with an index from 1 to *N* (equal to #Rows), increasing from the wrist to the elbow.

Grid cell dimensions were defined to match the stimulation electrode size, ensuring spatial correspondence between mapped sites and the effective stimulation area. In this work, electrode size was approximately 1.5 cm, and the minimum cell dimension was set accordingly. The longitudinal spacing was fixed at 2 cm, while the transverse spacing varied from 1.5 cm to approximately 2.5 cm to accommodate variations in forearm circumference. The maximum transverse spacing ($s_{e}$) at the elbow was computed using the proportional relationship $s_{e}=c_{e}s_{w}/c_{w}$, where $s_{w}$ is the transverse spacing at the wrist.

The longitudinal position of the grids was defined by placing the distal boundary at 15% of the $l_{fa}$ measured from the wrist midpoint. The transverse alignment was established by aligning the longitudinal boundaries between columns A–B and G–H with a reference axis connecting the middle finger to the elbow joint center (green lines in Figure 3).

**Figure 4:**
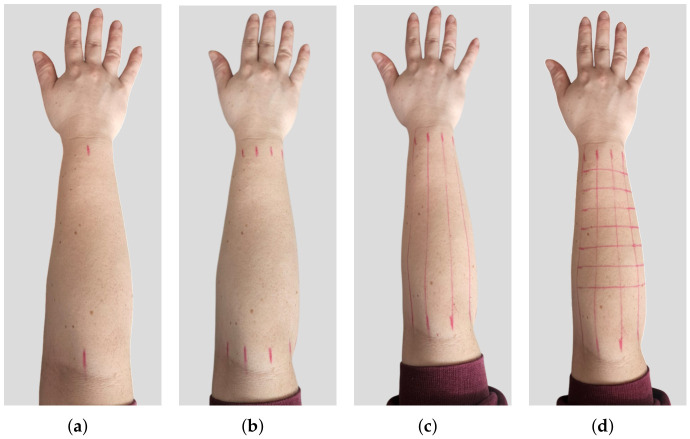
Grid construction procedure for the forearm. The grid is defined in four steps (illustrated here for the posterior side): (**a**) identification of wrist and elbow centers; (**b**) placement of equidistant marks along the centerline, extending once radially and three times ulnarly; (**c**) connection of marks to form the longitudinal columns; (**d**) completion of the grid by tracing transverse rows.

Figure 4 illustrates the four operative steps required to construct the posterior grid for finger extensor muscle stimulation:

- The subject is seated in front of a table and asked to extend the arm with the palm facing downward. In this position, the wrist and elbow midpoints are identified.
- Equidistant marks are placed along the centerline—spaced 1.5 cm at the wrist and 2.5 cm at the elbow—extending once toward the radial side and three times toward the ulnar side.
- The marks are connected to form the grid columns.
- The grid is completed by tracing rows at regular intervals of 2 cm, starting from the top boundary at 15% of the forearm length from the wrist and extending up to 75% of the forearm length.

The same procedure can be applied to construct the anterior grid by extending the arm with the palm facing upward.

### 2.2. Stimulation Sites Identification

To keep the protocol duration reasonable, the stimulation point search is performed using brief stimuli (200 ms) and predefined initial stimulation parameters. Parameters initialization is performed separately for the anterior and posterior grids, with the electrodes positioned in a central region of each grid. Stimuli with a fixed PW of 250 μs are delivered while progressively increasing the CI until a visible fingers response is observed. If no movement is detected when the CI reaches 12 mA, the test is restarted with a PW increased of 50 μs.

Once the initial parameters are identified, the search for stimulation sites is performed for each individual finger movement. Since varying the positions of two electrodes across the grid yields a number of configurations that is impractical to test within a reasonable time (e.g., 552 placements for a 6 × 4 grid), the proposed protocol keeps one electrode fixed (the return electrode), while the other (the exploring electrode) sweeps across the grid cells.

The default placement of the return electrode is located outside the main grid, adjacent to either the top or bottom boundary, within the gray-shaded regions shown in Figure 3 and indexed by lower-case letters. The specific placement depends on the stimulated finger: for TE movement, the return electrode is positioned in cell “a”; for other extension movements (IE, ME, RLE), it corresponds to cells “b”–“d”; for flexion movements (IF, MF, RLF), it corresponds to cells “e”–“g”. Each grid cell is then systematically tested with the exploring electrode using the initialized FES parameters to assess whether the stimulation elicits the desired finger movement.

**Figure 5:**
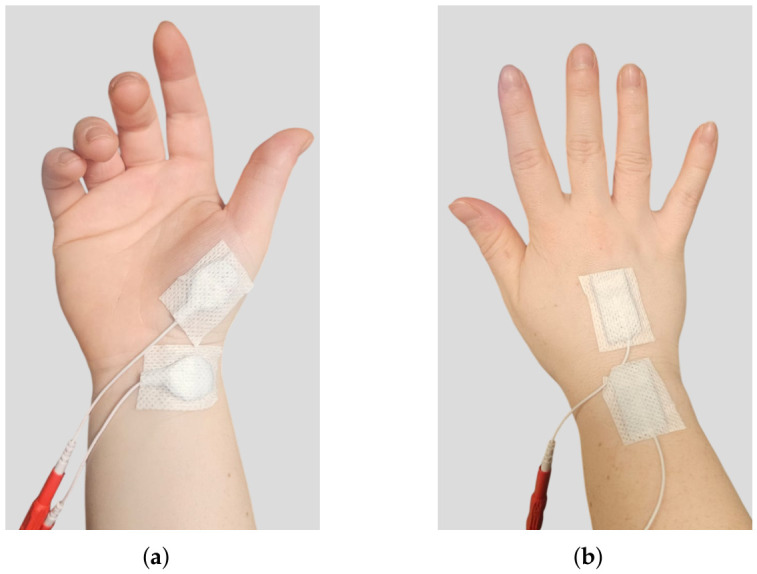
Special electrode placements. (**a**) Electrode configuration used for thumb opposition (TO). (**b**) Example of Takahashi’s electrode placement [23], with one electrode positioned on the dorsal side of the wrist and the other on the dorsal aspect of the hand to stimulate the interossei and lumbrical muscles. This configuration is shown for middle finger flexion.

This calibration phase is required for each individual finger movement except for TO, whose target muscle, the opponens pollicis, is a small intrinsic muscle that can be reliably identified and selectively activated (Figure 5a).

Since small variations in electrode positioning can elicit different movements, a key limitation of the optimal point identification phase is the restriction to discrete grid cells, excluding intermediate positions. Additionally, some electrode configurations may cause discomfort. To mitigate these issues, post hoc adaptations are introduced—such as testing intermediate positions or adjusting the fixed electrode—to improve subject comfort and avoid overlooking potentially effective stimulation sites.

Another adjustment concerns the placement of the fixed electrode. In some cases, a movement different from the intended one could be successfully elicited. If such a movement cannot be obtained using its default fixed electrode placement, the alternative configuration is considered valid. This approach introduces flexibility in the placement of the fixed electrode while keeping the number of iterations manageable. Moreover, allowing different movements to share the same fixed electrode position is not problematic, as these regions are larger than individual grid cells and can accommodate multiple electrodes.

If, at the end of the calibration phase, it is not possible to identify an effective stimulation site for some finger flexion movements, an alternative stimulation approach proposed by Takahashi et al. [23] is employed. In this case, a 1.5 mm × 1.9 mm rectangular electrode, is placed on the posterior side of the wrist, while the second electrode is positioned on the dorsal side of the hand to target the lumbrical and interossei intrinsic muscles, as depicted in Figure 5b. However, the resulting gestures are limited in quality, with flexion restricted to the metacarpophalangeal (MCP) joint.

### 2.3. Stimulation Parameters Calibration

The second phase of the calibration protocol aims to determine the optimal parameter set by individually tuning CI and PW to achieve efficient, comfortable, and subject-specific muscle activation. The CI is calibrated first by iterating a stimulation pattern consisting of a two-second activation phase followed by a four-second rest phase. While keeping the PW initialized previously, the CI is progressively increased from 4 mA in 2 mA steps, corresponding to the stimulator resolution [33], until either the desired movement is achieved or the subject reports discomfort. Upon successfully inducing the functional movement without discomfort, the protocol advances to the PW calibration phase.

The same stimulation pattern is then applied while keeping the optimal CI, and the PW is varied from −30% to +30% of the initial value in 10% steps. The PW search range and step size were selected to minimize calibration time while allowing effective parameter optimization. Lower PW values are tested to determine whether the functional movement can be elicited with reduced stimulation energy. Conversely, increasing the PW is often necessary to target deeper muscles [14,34]. The PW adjustment is terminated once the functional movement of the target finger is successfully achieved.

### 2.4. Dexterous Hand Movements Stimulation

The FES parameters and electrode layout identified for isolated movements can be combined to elicit dexterous hand movements or gestures. Moreover, the grid system presented in Section 2.1 enables rapid reproduction of the stimulation setup used during calibration in subsequent rehabilitation sessions.

However, after reconstructing the grids on the subjects’ forearms, the previously identified optimal stimulation points and parameters are re-evaluated to verify that the desired movements can still be effectively elicited. Slight variations in grid placement between sessions may require minor adjustments to electrode positioning to locate the optimal sites. In addition, stimulation parameters may vary across sessions, necessitating dedicated fine-tuning.

Once all finger movements have been successfully tested, the hand gesture stimulation phase begins. The subject is seated in front of a desk and asked to assume a neutral position, with the elbow resting on the desk surface and the forearm elevated. In this position, which replicates the posture typically adopted during everyday hand movements, the finger joints are relaxed, resulting in slight flexion. Moreover, this position facilitates the placement of multiple FES channels, minimizing movement obstruction caused by the electrode cables.

The stimulation pattern is designed to reproduce the physiological activation timing across different fingers. For example, for gestures requiring the thumb to oppose over the other fingers (i.e., Hand closed, One, and Two in Figure 2), activation of the TO channel is delivered with a 200 ms delay to ensure that thumb opposition occurs after finger flexion, allowing the thumb to be positioned over the flexed fingers. Preliminary tests indicated that this delay was sufficient to achieve the desired movement sequence.

## 3. Experimental Validation

To verify the feasibility of the proposed compositional calibration approach for eliciting dexterous hand gestures through multi-channel FES, an experimental validation was conducted involving 13 healthy volunteers (32.7±13.1 years).

Data collection was carried out at the Department of Electronics and Telecommunications, Politecnico di Torino (Torino, Italy). Prior to participation, each subject received detailed information regarding the study objectives and the safety of the experimental procedures. Written informed consent was obtained from all participants in accordance with protocol No. 445154, approved by the Comitato Bioetico di Ateneo of the Università degli Studi di Torino (Torino, Italy).

The validation was designed to reproduce the entire multi-channel FES protocol represented in Figure 1 and consisted of two separate sessions aimed at evaluating both the calibration/recovery procedure and the compositional generation of dexterous hand gestures. During the first session, all thirteen participants underwent the calibration protocol described in Section 2 to identify the FES parameters and electrode placements for the eight isolated movements. The second session was designed to evaluate both the recovery of previously calibrated stimulation sites and parameters after electrode removal and grid reconstruction, and the feasibility of generating multi-finger gestures through the composition of individual finger movements. Consequently, participation in this phase required the availability of the elementary movements necessary to synthesize the selected gesture set shown in Figure 2. The first eligibility criterion was the successful calibration of at least six of the eight isolated movements. Admission to the second session was further restricted to participants exhibiting a sufficiently complete and selective set of extension movement primitives, as these movements contributed to the majority of the tested gestures and no alternative strategy was available when they could not be elicited reliably. All experiments were conducted on the dominant forearm, which corresponded to the right side for all participants. At the end of each session, participants completed a feedback questionnaire to report their subjective experience of the stimulation.

Electrical stimulation was delivered using an 8-channel stimulator, the RehaStim2 (HASOMED^®^ GmbH, Magdeburg, Germany) [33], which offers high flexibility in configuring stimulation parameters. Stimulation was applied at a constant frequency of 40 Hz using FIAB PG479/32W surface electrodes (FIAB Spa, Vicchio, Italy) [35], trimmed to 19 mm × 16 mm to increase selectivity [24].

Stimulation parameters were controlled through a custom graphical user interface (GUI) developed in Python^®^ 3.10. The interface ensured correct execution of the experimental protocol by preventing configuration errors and eliminating the need for manual parameter adjustments across different protocol stages, while enabling structured storage of subject-specific data.

During both experimental sessions, successful elicitation of each movement was assessed in real time through visual inspection of the evoked motion by the operator, thereby reproducing the intended practical use scenario of the proposed framework. Moreover, the integration of an automated movement-assessment method was outside the scope of the present study. When a movement was deemed successfully elicited, a trial was recorded for subsequent quantitative analysis. As described in the next section, video-based hand kinematics were extracted post hoc to quantify movement excursions and individuation. These kinematic metrics were used exclusively to characterize the elicited movements and were not used to determine movement success.

### Hand Kinematics Analysis

While finger movements were assessed visually in real time, experimental trials were recorded via a smartphone and subsequently post-processed using the SAM 3D Body model [36] (3DB) to extract hand kinematics. The 3DB model estimates full-body pose from monocular images using a parametric representation known as the Momentum Human Rig [37]. Recent studies have demonstrated the applicability of 3DB for rehabilitation-oriented movement analysis using monocular RGB recordings, including the extraction of hand and upper-extremity kinematics [38,39].

**Figure 6:**
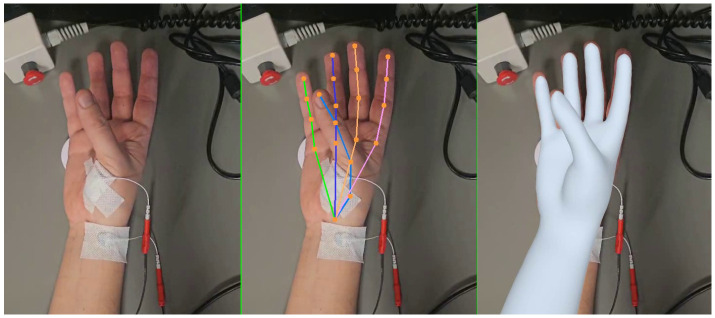
Example of hand pose estimation using the SAM 3D Body model (3DB). **Left**: Raw monocular image of a thumb opposition movement captured during a trial and used as input to the 3DB model. **Middle**: Detected 2D hand keypoints and skeletal structure overlaid on the input image. **Right**: Reconstructed Momentum Human Rig representation aligned with the input image.

As shown in Figure 6, the 3DB model produces both a surface mesh that captures the hand shape and gesture, and an underlying skeletal representation defined by 21 keypoints corresponding to joint centers and fingertip locations. The hand kinematic model comprises 16 joints with a total of 27 degrees of freedom, as summarized in Table 1. Each kinematic recording begins with the hand in a rest position and ends when electrical stimulation ceases. For each joint component *k* of the finger *i*, denoted $\theta_{ik}$, the mean angular displacement from the initial position is computed as

(1) $$A_{ik}=\frac{1}{T}\int_{0}^{T}\left(\theta_{ik}\left(t\right)-\theta_{ik}\left(0\right)\right)dt,$$

where *T* is the total duration of the recording.

The individuation index (II) was computed to quantify the degree of independence of movement of a target finger induced by electrical stimulation, relative to the non-target fingers [23]. This parameter assumes values closer to one as the movement of the target finger becomes more isolated, whereas it decreases—and may even become negative—as the degree of isolation from the non-target fingers diminishes.

In contrast to [23], where the II was computed using the absolute values of joint angular displacements, in this work the II formulation was designed to account for the expected direction of movement. Specifically, for certain movements, some joint components are expected to be either positive or negative. Accordingly, the II was computed using the signed $A_{ik}$ multiplied by the expected sign, so as to yield a positive contribution only when $A_{ik}$ occurs in the expected direction. Furthermore, since for some movements only a subset of joint components responded consistently to stimulation, the maximum value among the corrected excursions for the target finger, denoted $A_{i}^{*}$, was used. Conversely, for a non-target finger *j*, the maximum absolute displacement, $\left|A_{j}\right|$, is used. was considered. As a result, the individuation index for a set of target fingers Γ was computed as

(2) $$II_{\Gamma }=1-\frac{\frac{N_{t}}{5-N_{t}}\sum_{j\notin \Gamma }\left|A_{j}\right|}{\sum_{i\in \Gamma }A_{i}^{*}},\;\mathrm{where}\;N_{t}=\left|\Gamma \right|.$$

The magnitude of negative II values reflects the extent to which the excursions of non-target fingers exceed those of the target finger, with increasingly negative values indicating progressively poorer movement selectivity. Conversely, when non-target finger excursions are negligible, the II approaches one. It is worth noting that if the average behavior of the target finger is opposite to that expected (e.g., the finger flexes instead of extending), $II_{\Gamma }$ may assume values greater than one. However, in this condition the individuation measure is not meaningful; therefore, in practice, the index is considered undefined and is not computed.

Moreover, not all joint components of the target finger are equally informative for a given movement (e.g., index abduction during index extension stimulation). Erroneous stimulation sites or incidental passive dynamics may induce substantial excursions in non-target kinematic components. To ensure that the computation of $II_{\Gamma }$ reflects only the kinematics of interest, a preliminary analysis was conducted on the validation dataset to identify, for each target finger and stimulated movement, the informative kinematic variables along with their expected excursion signs. Among the 27 DoFs summarized in Table 1, the subset of kinematic variables identified as informative for the investigated movements is highlighted in gray.

## 4. Results and Discussion

### 4.1. Stimulation Sites and Parameters Calibration

**Figure 7:**
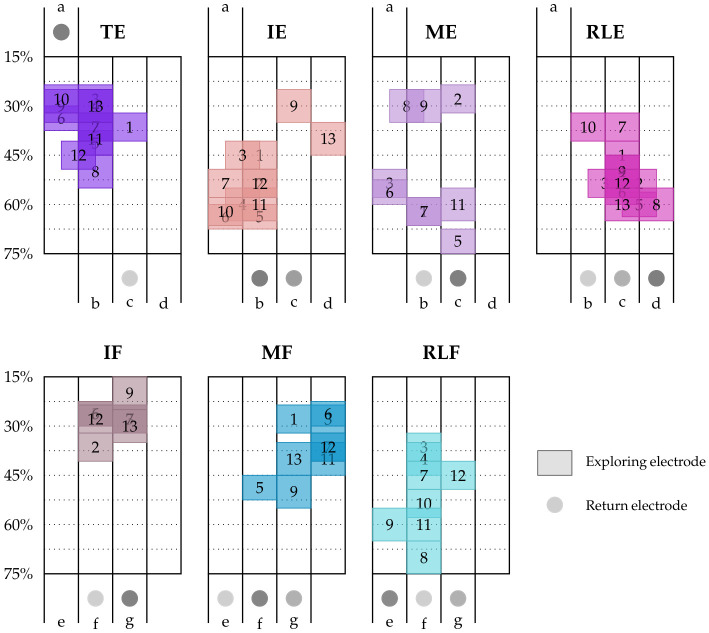
Optimal electrode configurations and corresponding stimulation distributions across subjects. **Top** and **bottom** panels represent electrode positioning grids for extension and flexion movements, respectively. Colored cells indicate selected stimulation sites, with numbers denoting individual subjects. The vertical position and extent of each subject’s stimulation sites are normalized to forearm length and expressed as a percentage. Gray circles indicate return electrode positions, with darker shading reflecting overlapping placements across subjects. Lowercase letters (a–g) index return electrode placements.

As illustrated in Figure 7, isolated finger movements are elicited with electrode placements targeting spatially distinct regions within the two electrode positioning grids. Depending on the participant anthropometry, the grid dimensions ranged from 6 × 4 to 8 × 4 cells (rows × columns), corresponding to 24–32 candidate stimulation locations per grid. For example, TE is elicited with exploring electrode placements targeting the top-left portion of the posterior grid (20–45% of the forearm length within the radial hemiplane). In contrast, RLE is elicited from the bottom-right region (45–65% within the ulnar hemiplane). By comparison, for IE and ME, effective electrode layouts are more spatially dispersed, spanning from the bottom-left (45–65% within the radial hemiplane) to more distal sites.

In the anterior grid (bottom panels of Figure 7), distinct regions associated with flexion movements are also observed. IF is elicited by stimulation of distal grid regions (central columns at 15–40% of the forearm length), whereas MF is elicited at slightly more proximal sites shifted toward the radial side. RLF, in contrast, is predominantly elicited from the central-ulnar region of the proximal grid. For all movements, return electrode placement predominantly corresponded to the column aligned with the target finger, with fewer exceptions in neighboring cells. Although a single optimal sensor placement cannot be identified across subjects, these results indicate that stimulation site selection can be constrained to specific grid regions based on the target finger and desired movement. This observation suggests that a generalized electrode placement strategy could be developed at the regional level. Rather than searching the entire grid, the exploration could be focused on the grid regions most frequently associated with each target movement, thereby improving scalability while preserving subject-specific customization.

**Figure 8:**
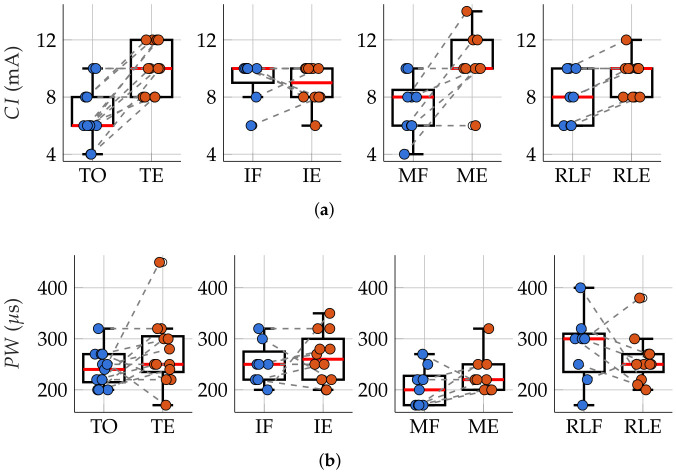
Calibrated FES parameters for eight isolated finger movements. (**a**) Current intensity (CI) and (**b**) pulse width (PW) for flexion/opposition (blue) and extension (orange). Each point represents an individual subject, with dashed lines indicating paired measurements. Boxplots show the median, interquartile range, and range. Movements are grouped by finger (thumb, index, middle, and ring/little). Points without a dashed connector indicate that the corresponding paired movement was not elicited.

As observed for the optimal electrode placements, the calibrated stimulation parameters also showed substantial inter-subject variability, with CI values ranging from 4 mA to 14 mA and PW values from 170 μs to 450 μs. Except for the index finger, extension movements required higher stimulation intensities than flexion movements (Figure 8a). ME exhibited the highest mean CI (10.4±2.1 mA), whereas IE showed the lowest (8.8±1.3 mA). In contrast, flexion movements and thumb opposition were associated with lower CI values, with the highest mean observed for IF (9.2±1.4 mA) and the lowest for TO (6.8±1.8 mA).

These findings suggest that extension movements require recruitment of a larger number of muscle fibers. This interpretation is further supported by the experimental setup, in which extension movements occur in the hyperextension range, where greater passive resistance is encountered. This constraint is less pronounced for the index finger, which is characterized by a broader range of motion. No clear differences in PW values were observed between flexion and extension movements (Figure 8b). However, middle finger movements exhibited lower PW values compared to the other fingers, suggesting that their activation may involve more superficial muscle fibers.

### 4.2. Isolated Finger Movements Stimulation

Table 2 reports the median and interquartile range of the signed mean angular displacement $A_{ik}$ obtained during the stimulation of isolated movements after the calibration phase. Overall, the highest median magnitudes are consistently observed in the joints of the target finger (blue frames) for each movement, indicating that the stimulation protocol was generally able to elicit selective activation of the intended finger. Moreover, the sign of the main excursions is consistent with the expected behavior of the intended movement: flexion movements are generally associated with positive values of the target joint kinematics along the flexion–extension axis (*z* components), whereas extension results in negative values.

Flexion angular displacements generally exhibit higher magnitudes than their extension counterparts, with primary involvement of the PIP joints (2z) in the former and the MCP joints (1z) in the latter. This discrepancy may be attributed to the greater range of motion available for flexion, whereas extension movements were more limited and primarily occurred within the hyperextension range.

For the TO movement, the $T_{1y}$ angle, associated with thumb abduction–adduction, exhibits the highest median displacement which, given its positive sign, suggests that thumb abduction at the CMC joint is the primary activation elicited by stimulation of the opponens pollicis. This primary activation is accompanied by slightly lower, yet still significant, thumb internal rotation and flexion, contributing to the overall thumb opposition. Conversely, for the TE movement, the primary activation is reflected by the negative displacement of the CMC angle $T_{1z}$, associated with extension. However, the magnitude of this median displacement is lower than that observed for TO and is comparable to that of non-target joint kinematics (e.g., $L_{1z}$). This discrepancy between the maximum angular displacements in TO and TE is likely attributable to the anatomical characteristics of the muscles involved: the opponens pollicis is a small intrinsic muscle that is relatively easy to target, whereas the extensor pollicis longus is located within the deep dorsal compartment of the forearm [40], making selective surface stimulation more challenging.

The IE movement was observed in all subjects except one, producing moderate but well-isolated excursions, whereas IF was more challenging to elicit, yielding higher angular displacements in the target finger but with a lower degree of isolation. This difference can be explained by the underlying muscle anatomy: index extension is primarily controlled by the extensor indicis, which enables strong and selective activation. In contrast, index flexion is mediated by the flexor digitorum communis, which also acts on the middle, ring, and little fingers, thereby making isolated activation more difficult.

Middle finger extension (ME) was the least frequently observed among extension movements (found in 9 of the 13 participants), whereas MF was the most frequently observed and prominent among flexion movements. Both MF and ME were well isolated when correctly targeted, except in some cases where middle finger movement was accompanied by ring finger flexion.

While the RLF and RLE movements require the combined flexion/extension of the ring and little fingers, the results in Table 2 show a tendency to elicit the activation in only one of the two, namely the ring finger for RLF and the little finger for RLE. The absence of little-finger flexion during RLF is consistent with previous observations [25]. Regarding RLE, the missing ring-finger extension may be related to the fact that ring and little finger extension rely on different extensor compartments, with ring finger extension primarily mediated by the extensor digitorum communis and little finger extension assisted by the extensor digiti minimi. Consequently, stimulation sites capable of eliciting little-finger extension may not necessarily produce a comparable activation of the ring finger. Moreover, ulnar deviation was often observed to follow little finger extension during RLE stimulation, likely due to the activation of the extensor carpi ulnaris, located on the posterior side of the forearm. In five subjects, the RLF movement could not be elicited due to unintended wrist flexion activation.

The analysis of the individuation index, II, across subjects and isolated movements, as summarized in Table 3, shows that, overall, successful stimulation produced well-isolated finger movements, except in the case of TE and RLE. In these successful cases, the maximum angular displacement of the target joint was typically more than twice the average displacement of the non-target fingers.

For flexion movements, the high II values are primarily driven by the larger angular displacements achieved by the target finger. In contrast, for extension movements such as IE and ME, high II values arise from better isolation of the target finger relative to the non-target fingers, despite generally lower angular displacement magnitudes compared to flexion movements.

TE and RLE exhibited the lowest II values, yielding negative in some subjects. For TE movements, the extremely limited thumb extension, combined with occasional movements of non-target fingers and episodes of radial deviation, contributed to the markedly low II values. Similarly, during RLE stimulation, the intended combined ring/little finger extension was often only partially achieved, with limited extension of the little finger and nearly absent displacement of the ring finger. This reduced the target movement term used in the computation of II, further lowering the index values. Moreover, in some cases, the ring finger exhibited slight passive flexion, likely induced by concomitant ulnar deviation (Subjects S3 and S4).

In all subjects excluding S4, S8, and S10, FES successfully elicited at least six of the eight isolated movements, with subjects S3, S7, and S9 achieving all targeted finger movements. However, in this work, additional criteria were applied before admitting subjects to the dexterous gesture stimulation session. Specifically, while missing flexion movements could be partially compensated using the Takahashi technique, no alternative strategy was available for absent extension movements. Consequently, subjects S12 and S13 were considered ineligible because middle finger extension could not be elicited, whereas subjects S5 and S7 were excluded because thumb extension exhibited insufficient selectivity for reliable gesture synthesis. In contrast, RLE was required in only two of the seven tested gestures and was therefore not considered an essential movement primitive for participation in the second session. For this reason, subjects exhibiting a missing or poorly isolated RLE movement could still be admitted to the gesture-composition phase. Of the six participants meeting the eligibility criteria, five (S2, S3, S6, S9, and S11) completed the second-session evaluation.

### 4.3. Dexterous Hand Gestures Stimulation

In this phase, the stimulation sites and parameters calibrated during the previous session enabled the generation of composite stimulation patterns through the linear combination of individual finger movements. However, inaccuracies in electrode grid reconstruction and subject-specific physiological variations made fine-tuning of electrode placement and FES parameters essential to achieve optimal stimulation. Notably, while PW values remained relatively consistent across sessions, CI values exhibited fluctuations of 2 mA to 4mA. These observations highlight that the proposed grid should be interpreted as a structured search and relocation framework rather than as a fully deterministic anatomical map, since its primary purpose is to facilitate the recovery of optimal electrode placement and stimulation parameters despite inter-session changes.

Five of the thirteen subjects (S2, S3, S6, S9, S11) from the first session were included in this session. These participants met the eligibility criteria defined in Section 3, namely the successful elicitation of a sufficiently complete and selective set of extension movement primitives required for the synthesis of the selected gesture set. All seven selected gestures (Figure 2) were performed, except for S11, for whom the RLE movement was absent and could not be elicited. Consequently, the Four and Hand Open gestures could not be performed.

As shown in Table 4, the combined FES patterns were able to elicit the desired dexterous movements on average, with joint angle excursion signs consistent with the expected directions of finger motion. This agreement is particularly evident in primary joints associated with individual finger movements (Table 2), supporting the feasibility of the linear combination strategy.

In terms of magnitude, larger excursions are observed in the flexion/extension components of proximal joints driving the gestures, such as CMC, MCP, and PIP joints (i.e., 1z and 2z), whereas more distal joints (i.e., 3z) generally exhibit either smaller excursions or an higher variability. Notably, MCP flexion and PIP extension exhibited higher excursion magnitudes compared to isolated movements. This may be partly explained by the reduced number of subjects included in this analysis, as well as by the different resting position (i.e., neutral pose), which enabled a greater range of motion, particularly for extension.

Despite the overall encouraging results supporting the possibility of eliciting combined finger movements, qualitative observations revealed some inaccuracies in movement execution. Finger flexion was the primary source of the poorest performance, particularly when using the alternative stimulation approach (Takahashi’s method). For instance, Subject S2, who did not exhibit distinct MF and RLF movements, showed reduced performance when these flexions were required (i.e., One, Hand Close, and Thumb Up). In contrast, Subjects S3 and S9, who exhibited all expected sub-movements, achieved the highest-quality execution of complex gestures.

In approximately half of the trials, wrist movements were inadvertently elicited to a significant extent. These were typically observed in gestures involving the extension of more than two fingers, particularly those engaging the thumb and the ring/little fingers (e.g., Three, Four, and Hand Open). This behavior may reflect limited selectivity of multichannel stimulation, whereby current spread leads to co-activation of muscles responsible for wrist extension as well as ulnar and radial deviation. Notably, for some subjects (particularly S3 and S11), the wrist appeared to be more readily recruited.

### 4.4. Operational Burden and Participants Feedback

From an operational perspective, the first session required approximately 2 h per participant, corresponding to an average of 15 min per target movement. The second session lasted approximately 1.5 h, including grid reconstruction, parameter fine-tuning, and stimulation of the seven gestures, corresponding to a reduction of approximately 25% in session duration compared to the initial calibration procedure.

The questionnaire showed that most participants (11 out of 13) described the stimulation as a light vibration, while the others reported a mix of vibration and burning depending on electrode placement. Four participants reported some pain or discomfort. However, all participants rated the overall tolerability as 3 or higher on a 5-point scale. Discomfort was primarily attributed to the positioning required to identify stimulation points during finger flexion tasks. No participants reported difficulty following the protocol or feeling fatigued after completing the tasks, and only one participant felt that the first two parts of the experiment were too long.

### 4.5. Limitations and Future Work

Despite the promising results supporting this compositional strategy, the experimental validation also highlighted several limitations that need to be further investigated and addressed to improve the proposed approach. Eight stimulation channels were insufficient to elicit coordinated movements of the ring and little fingers. The use of a 10-channel stimulator could enable full decoupling of all fingers while keeping system complexity manageable.

It should be noted that the gesture-composition analysis was performed only on participants exhibiting the movement primitives required to synthesize the selected gesture set. Consequently, these results should be interpreted as a feasibility evaluation of the proposed compositional framework rather than as an estimate of gesture-generation success rates in whole population.

Stimulation outcomes during calibration were assessed qualitatively, consistent with the study’s feasibility-oriented scope. Future work will investigate the integration of real-time kinematic feedback of finger movements with automated evaluation frameworks to enable efficient identification of stimulation sites and optimization of parameters, while preserving therapist oversight and patient involvement.

Multi-channel stimulation of the forearm frequently resulted in substantial wrist activation, even when individual sub-movements did not initially elicit wrist involvement. One possible solution is to include an additional stimulation channel dedicated to wrist stabilization.

The time required to identify stimulation sites could be reduced by restricting the search space to the grid regions highlighted by the heatmaps in Figure 7. Furthermore, reducing the exploration space for active electrode placement would allow a more extensive exploration of return electrode configurations, facilitating the identification of optimal stimulation sites while maintaining a manageable number of electrode placement combinations.

The present study was conducted exclusively on healthy participants. Although the proposed framework relies on subject-specific calibration and is therefore not restricted to a particular condition, future studies should investigate its applicability in populations affected by neuromuscular impairments, where the achievable movement selectivity and calibration outcomes may differ substantially from those observed in healthy subjects.

This research also highlights several directions for improving the proposed protocol. First, grid placement could be enhanced through the use of 3D scanning and augmented reality to ensure accurate and repeatable electrode positioning without manual intervention. Second, future work should investigate the stimulation of more complex and functional hand gestures, such as grasp-and-release and pinching.

Building on our research on FES control and EMG-based hand-gesture recognition [41,42,43], the proposed calibration framework facilitates future investigations into the use of muscular activity for designing multi-channel stimulation patterns [44], thereby extending bio-inspired methods to hand rehabilitation.

## 5. Conclusions

This study presented a compositional calibration framework for multi-channel functional electrical stimulation, enabling the personalization of stimulation for isolated finger movements and their combination into functional hand gestures. The proposed approach reduces calibration complexity by relying on a structured spatial grid and a limited number of stimulation channels.

Experimental results demonstrated that most participants were able to achieve multiple isolated finger movements with good selectivity, and that these movements can be combined to generate a set of functional hand gestures. However, the results also highlight a trade-off between selectivity and compositionality, as simultaneous multi-channel stimulation may introduce co-activation effects that reduce movement quality.

These findings suggest that compositional calibration represents a viable strategy for simplifying the configuration of FES systems while enabling flexible generation of multi-finger movements. At the same time, they indicate that scaling this approach to more complex or highly dexterous hand functions may require additional control strategies or feedback mechanisms to mitigate channel interactions.

Future work will focus on validating the proposed framework in clinical populations and integrating adaptive or closed-loop control methods to improve robustness and usability in real-world rehabilitation scenarios. 

## Figures and Tables

**Table 1:** Mapping of SAM 3D Body (3DB) hand kinematics to anatomical joints and associated degrees of freedom (DoF). Gray shading highlights the kinematic variables identified as informative for the investigated movements. CMC = carpometacarpal; MCP = metacarpophalangeal; PIP = proximal interphalangeal; DIP = distal interphalangeal; IP = interphalangeal joint.

	Wrist	CMC	MCP	PIP	DIP	IP
Thumb	$T_{0y}$, $T_{0z}$	$T_{1x}$, $T_{1y}$, $T_{1z}$	$T_{2z}$	-	-	$T_{3z}$
Index	-	-	$I_{1x}$, $I_{1y}$, $I_{1z}$	$I_{2z}$	$I_{3z}$	-
Middle	-	-	$M_{1x}$, $M_{1y}$, $M_{1z}$	$M_{2z}$	$M_{3z}$	-
Ring	-	-	$R_{1x}$, $R_{1y}$, $R_{1z}$	$R_{2z}$	$R_{3z}$	-
Little	-	-	$L_{1x}$, $L_{1y}$, $L_{1z}$	$L_{2z}$	$L_{3z}$	-

Internal (+)/External (−) rotation, Abduction (+)/Adduction (−), Flexion (+)/Extension (−).

**Table 2:**
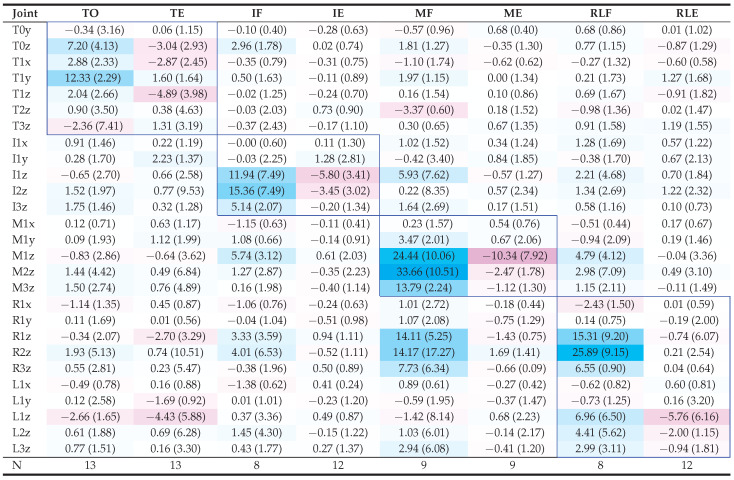
Median (IQR) signed mean angular displacements $A_{ik}$ for each joint during isolated finger stimulation. Columns correspond to target movements and rows to joint kinematics. Blue frames highlight the joint components associated with the target finger for each movement (T: thumb, I: index, M: middle, R: ring, L: little). N indicates the number of subjects with successful elicitation. Cell shading intensity reflects the magnitude of the median value; cyan shading denotes positive angular displacements, whereas magenta shading denotes negative angular displacements.

**Table 3:**
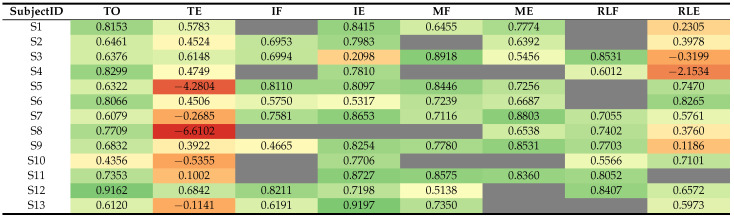
The heatmap displays Individuation Index (II) values across subjects and elicited movements. Cells are colored along a continuous scale from red (poorly isolated movements) to green (well-isolated movements), while gray cells indicate missing movements.

**Table 4:**
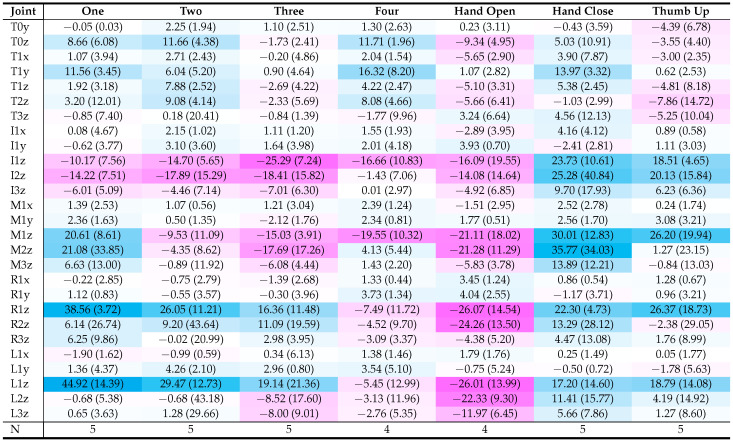
Median (IQR) signed mean angular displacements $A_{ik}$ for each joint during dexterous gesture stimulation. Columns correspond to the target gestures, while rows represent joint kinematic components (T: thumb, I: index, M: middle, R: ring, L: little). N denotes the number of subjects in whom each gesture was successfully elicited. Cell shading intensity reflects the magnitude of the median value; cyan shading denotes positive angular displacements, whereas magenta shading denotes negative angular displacements.

## Data Availability

The raw data supporting the conclusions of this article will be made available by the authors on request.

## Abbreviations

The following abbreviations are used in this manuscript:

FES	Functional Electrical Stimulation
CI	Current Intensity
PW	Pulse Width
II	Individuation Index
MCP	Metacarpophalangeal joint
PIP	Proximal Interphalangeal joint
DIP	Distal Interphalangeal joint
IP	Interphalangeal joint
CMC	Carpometacarpal joint
IE	Index Extension
IF	Index Flexion
ME	Middle Extension
MF	Middle Flexion
RLE	Ring and Little Extension
RLF	Ring and Little Flexion
TE	Thumb Extension
TO	Thumb Opposition
